# Evaluation of the Limb Symmetry Index: The Side Hop Test

**DOI:** 10.3389/fphys.2022.874632

**Published:** 2022-06-17

**Authors:** Sanja V. Mirković, Saša Đurić, Vedrana Sember, Olivera M. Knezevic, Maja Pajek, Milan M. Mirković, Dragan M. Mirkov

**Affiliations:** ^1^ University of Belgrade, Faculty of Sport and Physical Education, Belgrade, Serbia; ^2^ Liberal Arts Department—General Education, American University of the Middle East, Kuwait City, Kuwait; ^3^ Faculty of Sport, University of Ljubljana, Ljubljana, Slovenia; ^4^ University of Belgrade, Faculty of Medicine, Institute for Orthopaedic Surgery “Banjica”, Belgrade, Serbia

**Keywords:** side hop test, reliability, concurrent validity, limb symmetry index, stopwatch

## Abstract

The main objective of present study was to evaluate inter-rater reliability and concurrent validity of Side Hop Test stopwatch vs. force plates timing, and to determine the number of sessions and trials required to minimize the effects of learning on Side Hop Test total time and limb symmetry index. Fifteen healthy male physical education students (mean ± SD: age, 23 ± 3 years; height, 181 ± 9 cm; and weight 72 ± 6 kg) participated. Side Hop Test total time (stopwatch and force plates) of left and right leg, and limb symmetry index (force plates) were obtained over seven sessions conducted 5–7 days apart. Time recordings of two raters were similar (*t* = −0.56, *p* > 0.05) with high reliability (all ICC >0.99 and CV% <0.1) and no systematic bias when compared to force plate data (*p* > 0.05; for rater 1 and 2, respectively). Total time improved across the Sessions (*F* = 25.87, *p* < 0.01, *ω* ^2^ = 0.18) and Trials (*F* = 68.15, *p* < 0.01, *ω* ^2^ = 0.10), with no significant interaction between factors. No between-leg differences were detected (*F* = 0.52, *p* > 0.05, *ω* ^2^ = 0.001). Limb symmetry index ranged from 0.999 to 1.055 across all sessions and trials (all *p* > 0.05 and *ω* ^2^ < 0.00). Due to low coefficient of correlation, high interclass correlation coefficient, and the lack in heteroscedasticity, stopwatch measurements are valid to measure total time in the Side Hop Test. Moreover, stopwatch measurements could be reliably used to measure total time in the Side Hop Test, while the test could be administrated with only one experienced rater. Unlike total times, findings on limb symmetry index suggest it could be reliably assessed after seven familiarization sessions.

## Introduction

Functional asymmetries in the lower limbs are defined as a consistent task discrepancy between dominant and nondominant limbs (Sadeghi et al., 2000). They are determined by strength deficits between the two limbs (Fousekis et al., 2010) and are distinct from muscular imbalances, which represent a change in the force relationship between agonist and antagonist muscle pairs (Schlumberger et al., 2006; Jones and Bampouras, 2010). Functional asymmetries in the lower limbs have been the subject of numerous recent studies in many different contact, limited-contact and non-contact sports aimed at understanding the role of training in performance and in injury prevention (Lawson et al., 2006; Impellizzeri et al., 2007; Fousekis et al., 2010). Various classifications have been established for quantifying lower limb asymmetries including dominant opposed to non-dominant (Rouissi et al., 2016), stronger or weaker (Sato and Heise, 2012), left or right (Atkins et al., 2016), resulting in inconsistent methods for quantifying lower limb asymmetries with the exception of reporting these asymmetries as a percentage difference from one limb in respect to the other (Bishop et al., 2018). Thus, improving our understanding about the lower limb asymmetries might contribute to reducing the likelihood of getting injured (Croisier et al., 2008; Kiesel et al., 2014) and the related performance loss (Coratella et al., 2018; Maloney, 2019).

A functional performance test, classified as a performance-based measurement (Fitzgerald et al., 2001), is a useful measure in rehabilitation or condition assessment (Yoshida et al., 2011). One of the useful functional performance tests to assess lower limb function is the Side Hop Test (SHT), which can be used in both clinical and research settings in healthy and injured subjects (Kamonseki et al., 2018). In research settings, it has been mainly used to identify functional changes in patients with ankle instability (Martin et al., 2013; Scinicarelli et al., 2021; Wang et al., 2021) or after lower limb injuries (Ortiz et al., 2011).

Although the SHT is one of the most widely used functional test to assess ankle stability and has undergone the most research (Ortiz et al., 2005) in relation to lower limb functional performance (Gustavsson et al., 2006), there is still a lack of knowledge addressing the issue of the measurement characteristics of SHT. Stopwatches are up to date the most commonly used to measure the SHT performance (Docherty et al., 2005; Ortiz et al., 2005; Ortiz et al., 2011; Yoshida et al., 2011; Yoshida et al., 2018; Kamonseki et al., 2018), the measurement characteristics of mentioned test could be questionable. Alternative to stopwatch could be the contact matt which allows not only more reliable measures (Garcia-Lopez et al., 2005) but also the measurement of duration of every single phase of SHT, i.e., medial and lateral contact and “flight” times. To date, only six studies have investigated the reliability of SHT, four of which were conducted in adults (Caffrey et al., 2009; Wikstrom et al., 2009; Kockum and Heijne, 2015; Markström et al., 2018), one in young adult women (Ortiz et al., 2005), and one in children (Kamonseki et al., 2018). Markström with colleagues evaluated the within-session, test-retest reliability and agreement in trunk, hip, knee moments and ligament reconstructed persons and healthy-knee controls. They found excellent within-session reliability for angles in both groups (ICC >0.90) and excellent-to-good within-session reliability for moments (ICC >0.80), poor reliability for knee rotation (ICC <0.40) and excellent to fair test-retest reliability results for all angles and moments (ICC 0.47–0.91). Only Kockum and Heijne (2015) used a modified protocol that also assessed the number of repetitions for a given time, where all commonly used hop tests, including SHT, showed good to excellent intra-class correlation (0.84 < ICC >0.98), whereas Ortiz et al. (2005) applied inter-rater reliability measured with the stopwatch in a female population. The SHT showed good trial-to-trial and inter-rater reliability (ICC ≥0.87) but lower day-to-day reliability (ICC = 0.48), indicating consistency in a single session and inconsistency over time (Ortiz et al., 2005). However, the procedure they used had several limitations. All athletes were tested during the first week of training; therefore, training-related changes may have occurred. In addition, the authors (Ortiz et al., 2005) suggest re-measuring SHT with shorter time intervals between sessions to reduce the possibility of a learning effect. Moreover, Chan et al. (2020) established that for strength testing the number of familiarization sessions required for dominant and non-dominant limb required a similar number of sets (3). To our knowledge, none of the studies have examined the inter-rater reliability, concurrent validity, and number of familiarization sessions to minimize the learning effect of SHT in adult males.

Therefore, the main aim of the present study was to evaluate: 1) inter-rater reliability (two experienced raters) and concurrent validity [stopwatch SHT total time (TT) measurements, compared to TT derived from force plate measurements]; 2) determination of the number of familiarization sessions and trials required to minimize the effects of learning on SHT TT and limb symmetry index (LSI). We believe that our methodologically improved study will confirm the assumptions made in previous studies that stopwatch measurements could be a valid and reliable measure of both TT and LSI index. In addition, we hypothesized that although the complexity of the task in the side hop test would result in prolonged familiarization through multiple sessions and trials, the derived limb symmetry index would be stable across sessions and trials.

## Materials and Methods

### Participants

A sample of 15 male subjects (mean ± SD: age, 23 ± 3 years; height, 181 ± 9 cm; and weight 72 ± 6 kg) were physical education students. They were active during their normal academic curriculum, which included six to eight activity classes per week, with both low- and high-intensity exercises that did not include strenuous lower-body and leg exercises. They were excluded from the study if they were active athletes, had chronic medical conditions, cardiac problems, mechanical instability of the ankle joint, a history of orthopaedic surgery, a fracture of a lower extremity, or a history of an ankle sprain within 6 months. Lower limb dominance was determined as the side of preference to kick a ball (Seeley et al., 2008; Kamonseki et al., 2016). Subjects were instructed to avoid any strenuous exercise during the study. They were informed of potential risks and the purpose of the study and signed a written informed consent form that was in accordance with the Declaration of Helsinki and was approved by Institutional Review Board (02 No. 1307-2).

### Testing Procedure

The main task was to perform the side-hop test, which was performed as described earlier (Itoh et al., 1998) but with some modifications. The definition of the side-hop test was also similar to previous studies (Itoh et al., 1998; Demeritt et al., 2002; Gustavsson et al., 2006). The subject stood on the test leg and then jumped from side to side as quickly as possible between two parallel lines 30 cm apart, a total of 28 times or 14 cycles. A cycle is when the participant jumps laterally and medially back to the starting position. The first session was performed with the right leg and the first jump was always performed to the lateral side. The participants performed the side-hop test wearing athletic shoes.

### Experimental Protocol

**FIGURE 1:**
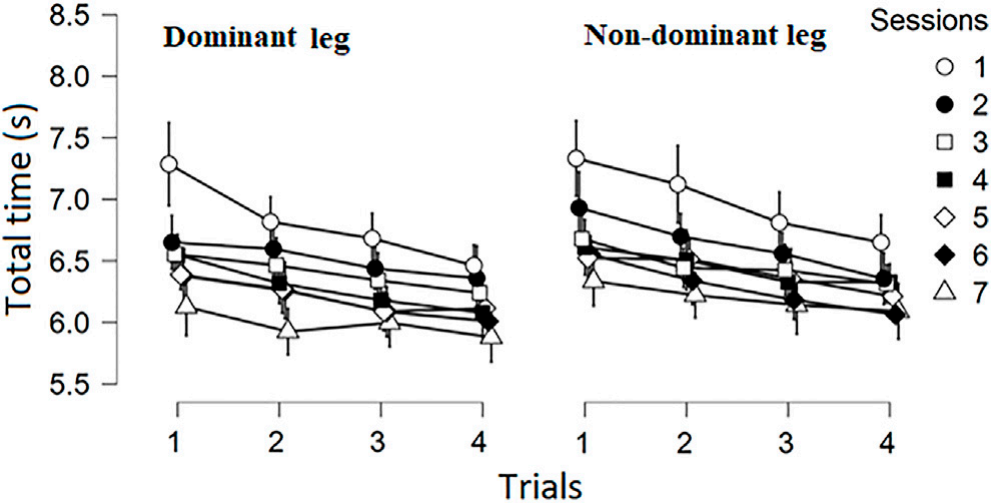
Flowchart of the experimental protocol.

Each participant completed seven sessions separated by a rest period of at least 5–7 days. The first three sessions were familiarization sessions and were measured with stopwatches only (SW; see Figure 1 for illustration). Two experienced researchers simultaneously measured the TT of the test. Each session was preceded by a standard warm-up procedure (5 min of cycling and 5 min of callisthenic and dynamic stretching). Prior to data collection, participants were instructed on how to perform the side-hop test and were allowed to practice the test twice (once with each leg). The trial was recorded as successful if subjects were able to complete the 28-jump repetitions, stepping on the line or having their untested leg touching the floor less than 3 times. Each subject made four trials with the right leg and four trials with the left leg, and if there were unsuccessful trials, the tests were cancelled and the subject retried. Subjects were allowed to rest for at least 3–5 min between all trials to avoid fatigue. Note that of the 28 jumps performed within each trial, the time was measured between the 4th and 24th jump (10 cycles), excluding the first four and last four jumps to prevent the effect of a poor start or finish to the trial.

### Experimental Procedures

Body height was measured using a standard anthropometer while body mass and percent body fat were assessed by a bioelectric impedance method scale (In Body 720; United States).

**FIGURE 2:**
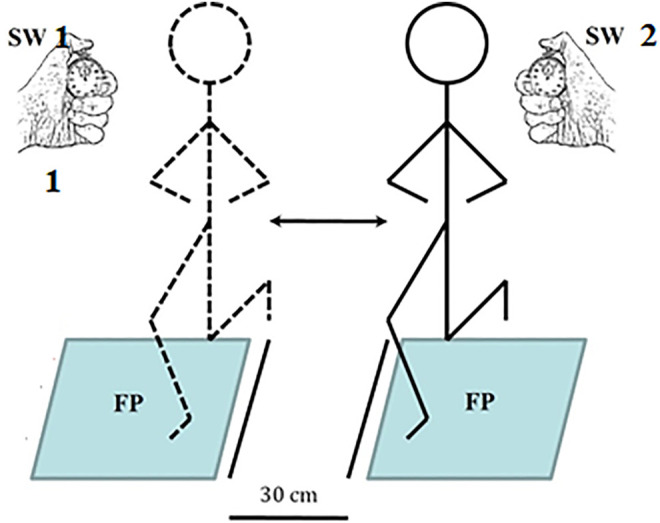
Illustration of the side-hop test performance and measurement of total time with FP and SW.

The time required to perform each trial was measured to the nearest 0.01 s using two handheld stopwatches (SW) and two synchronized force plates (FP; AMTI, BP600400; United States; see Figure 2 for illustration). Prior to data collection, subjects were instructed on how to perform the test and were allowed to perform the test as practice.

### Data Processing and Analyses

The force plate (AMTI, Inc., Newton, MA, United States; sampling frequency 1,000 Hz) was mounted and calibrated according to the manufacturer’s instructions. Custom-developed software (LabVIEW, National Instruments, version 10.0, Austin, TX, United States) was used for off-line processing of the vertical component of the ground reaction force (F). The data were low-pass filtered with a recursive second-order Butterworth filter with a cut-off frequency of 10 Hz. The total time from the beginning of the force signal in the 4th jump (landing medially) to the end of the 24th jump (landing in the starting position also medially and completing the 10th cycle) was measured.

### Statistical Analyses

The degree of agreement between total time measured by the raters (TTR1 and TTR2) and total time (TTFP) derived from force plate measurements was assessed through the measures of absolute [Coefficient of Variation–CV (%)] and relative (Intra-Class Correlation Coefficient—ICC) reliability (Hopkins, 2000). The systematic bias between the measurements was assessed through *t*-test for dependent samples. Also, limits of agreement- LoA = 95% using the Bland-Altman method were calculated and graphically presented (plotting the differences between each rater, and between each rater and the force plate, against the corresponding mean values (Martin Bland and Altman, 1986).

To explore the potential between sessions and trials’ learning effects on TT achieved with dominant/non-dominant leg, a three-way ANOVA (Leg as a between factor and Sessions and Trials as within factors) was applied. Two-way (Sessions and Trials as within factors) ANOVA on LSI was applied. When significant effects and their interactions were obtained, contrast analysis was applied. In addition, effect size was used to estimate the magnitude of differences of main effects, their interactions and contrast differences [*ω* ^2^ for ANOVA and Cohens’ d for Contrasts; (Field, 2018)]. The differences were considered as either small (*ω* ^2^ = 0.01; *d* = 0.2), moderate (*ω* ^2^ = 0.06; *d* = 0.5), or large [*ω* ^2^ = 0.14; *d* = 0.8; (Cohen, 1992; Kirk, 1996)]. The level of confidence (alfa) was set to 0.05.

## Results

### Interrater Reliability and Concurrent Validity of the Total Time Measures

**FIGURE 3:**
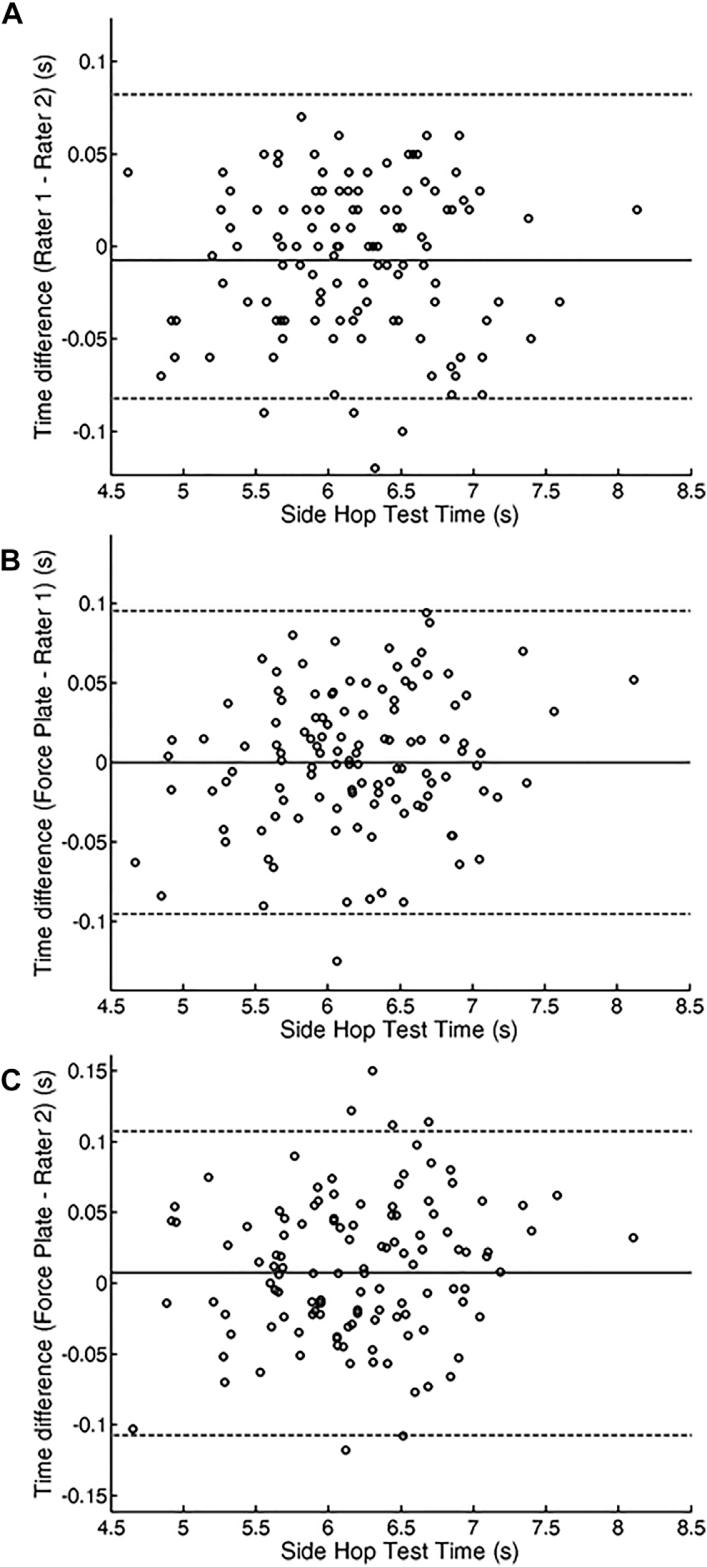
Bland–Altman plots for **(A)** interrater reliability showing the mean difference between raters rater (rater 2–rater 1). **(B)** Time measurement validity in rater 1 and **(C)** Time measurement validity in rater 2. The central solid line represents the mean differences (systematic error). The upper and lower dotted lines represent the upper and lower 95% limits of agreement (mean differences 6 1.96 SDs of the differences), respectively.

Components of inter-rater reliability of the TT measures, presented in Table 1 and in Figure 3A revealed high reliability, whereas the *t*-test revealed no differences in time recordings (i.e., no systematic bias) between rater one and two [t (29) = −0.56, *p* > 0.05].

**TABLE 1:** Inter-rater reliability (Rater 1 vs. Rater 2) and concurrent Validity (Rater 1 vs. Force Plate and Rater 2 vs. Force Plate) of time measures (total time in s) statistics for Side-hop test.

Total time (s)	Mean (SD) 1	Mean (SD) 2	Mean difference (LoC 95%)	CV (%)	ICC (95%CI)
Rater 1 vs. Rater 2	6.23 (0.62)	6.24 (0.63)	0.005 (−0.011 ÷ 0.022)	0.059	0.997 (0.994 ÷ 0.998)
Rater 1 vs. Force Plate	6.23 (0.62)	6.23 (0.61)	−0.001 (−0.021 ÷ 0.020)	0.075	0.994 (0.988 ÷ 0.997)
Rater 2 vs. Force Plate	6.24 (0.63)	6.23 (0.61)	−0.006 (−0.027 ÷ 0.015)	0.078	0.993 (0.988 ÷ 0.996)

*p < 0.05; SD, standard deviation; LoC, limit of confidence; CV, coefficient of variation; ICC, intraclass correlation coefficient.

The validity of the TT measures in the SHT when simultaneously measured by the two experienced raters and using force plate are shown in Table 1 and Figures 3B,C. CV and ICC revealed high reliability with no systematic bias when measurement outcomes of both raters were compared with corresponding data derived from force plate recordings [t (29) = −0.56, *p* > 0.05; t (29) = 0.47, *p* > 0.05; for rater one and two, respectively].

### Between-Session and Between-Trial Differences in Total Time Measurements and Limb Symmetry Index From Side Hop Test

**FIGURE 4:**
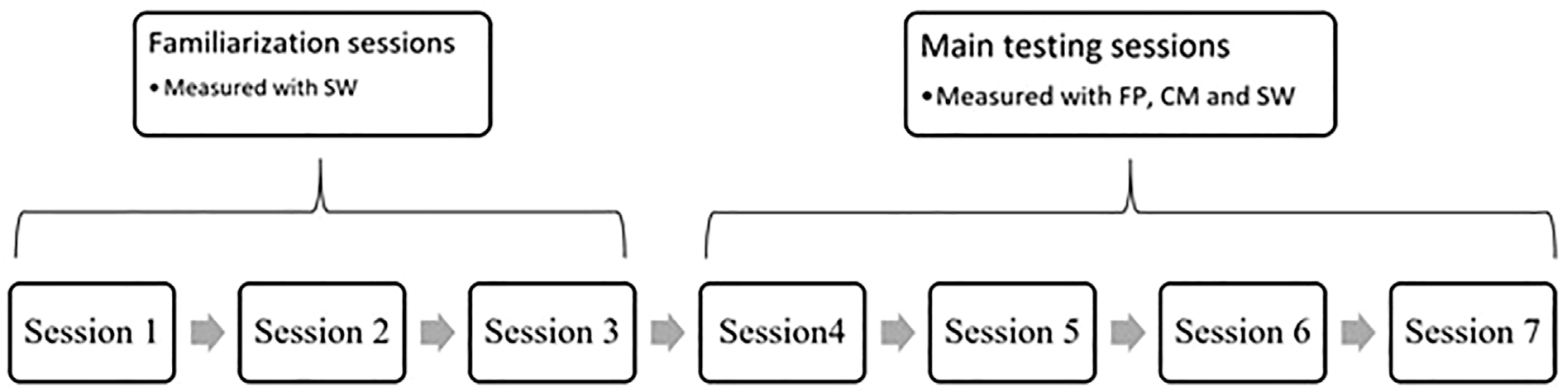
Strength assessed through 1RM (in kg).

Descriptive statistics for TT obtained across the sessions and trials for both legs are depicted in the Figure 4 (means presented as dots and SD as vertical error bars).

The Tree-way ANOVA revealed significant main effects of Sessions [F (3.6, 72.6) = 25.87, *p* < 0.01, *ω* ^2^ = 0.18] and Trials [F (3, 60) = 68.15, *p* < 0.01, *ω* ^2^ = 0.10], but not interactions. No between leg differences were detected [F (1, 20) = 0.52, *p* > 0.05, *ω* ^2^ = 0.001]. Further investigation of the change in TT between consecutive sessions (averaged for legs and trials) and between trials (averaged for legs and sessions) revealed that decrease in TT across the Sessions ended after second session (Table 2). However, regardless of the session and leg, contrast analysis for repeated measures, indicated high differences between all consecutive trials (Table 2).

**TABLE 2:** Results of Contrast analysis for repeated measures of total time (between-sessions and between trial differences).

		Mean difference (95% CI)	*t*-value (*df* = 120)	Cohen’s d
Session	1–2	0.321 (0.178 ÷ 0.465)	4.433*	1.00
	2–3	0.141 (−0.003 ÷ 0.284)	1.942	0.56
	3–4	0.070 (−0.074 ÷ 0.214)	0.965	0.26
	4–5	0.054 (−0.089 ÷ 0.198)	0.751	0.18
	5–6	0.074 (−0.069 ÷ 0.218)	1.024	0.34
	6–7	0.144 (0.001 ÷ 0.287)	1.982	0.52
Trials	1–2	0.171 (0.111 ÷ 0.232)	5.654**	1.215
	2–3	0.134 (0.073 ÷ 0.194)	4.409**	1.048
	3–4	0.104 (0.044 ÷ 0.165)	3.449**	1.122

*p < 0.05; **p < 0.01

Descriptive statistics for LSI [Mean (SD)] calculated across the sessions and trials are presented in Table 3. LSI ranged from 0.999 to 1.055 across all sessions and trials. The Two-way ANOVA revealed no significant main effects of Sessions [F (6, 60) = 0.712, *p* > 0.05, *ω* ^2^ = 0.001] and Trials [F (3, 20) = 0.263, *p* > 0.05, *ω* ^2^ = 0.001].

**TABLE 3:** Descriptive statistics for Limb symmetry index—LSI [Mean (SD)] calculated across the sessions and trials.

LSI mean (SD)		Trials			
		1	2	3	4
Sessions	1	1.013 (0.106)	1.045 (0.044)	1.020 (0.040)	1.032 (0.072)
	2	1.043 (0.032)	1.016 (0.044)	1.021 (0.052)	1.000 (0.042)
	3	1.020 (0.054)	0.999 (0.060)	1.016 (0.059)	1.013 (0.058)
	4	1.011 (0.049)	1.032 (0.086)	1.027 (0.078)	1.045 (0.070)
	5	1.025 (0.067)	1.041 (0.073)	1.047 (0.072)	1.019 (0.065)
	6	1.030 (0.064)	1.016 (0.067)	1.020 (0.073)	1.011 (0.057)
	7	1.038 (0.063)	1.054 (0.065)	1.027 (0.068)	1.039 (0.076)

## Discussion

Within the current study we have evaluated the inter-rater reliability and the concurrent validity of the TT measurements obtained from SHT when measurements were taken using stop watch and force plates. Furthermore, we aimed to determine minimum number of familiarization sessions and trials required to minimize the effects of learning on SHT, TT, and LSI. Our findings confirmed that SW measurements could be a valid and reliable measure of both TT and LSI index. In addition, we confirmed our hypothesis that complexity of the task in the side hop test results in prolonged familiarization through multiple sessions and trials. However, in contrast to the TT, the derived LSI was stable across both sessions and trials.

The first finding of the study indicated almost identical measures recorded by both raters and the records derived from the platform. In addition, exceptionally low CV, high ICC, and the lack in heteroscedasticity indicated high inter-rater reliability and concurrent validity of time measures, revealing that after a familiarization with the test protocol and with the measurement procedure, practitioners should be capable to make a reliable assessment of SHT. Although inter-rater reliability presents and important practical issue in applying test in praxis, surprisingly, this is the first study exploring the precision of SW measures of time needed to complete given number of jumps in the SHT. In their study aimed to evaluate reliability, measurement error, and construct validity of the SHT in male children and adolescents Kamonseki et al. (2018) have reported comparison of measurements obtained from two raters each recording one trial. As these two measurements were affected with both between-rater and between-trials variability, conclusion regarding the mutual comparison of raters outcome within the same session was not possible. Other studies on reliability and validity of stopwatch measures in some running field tests (Hetzler et al., 2008; Mann et al., 2015) or balance tests (Botolfsen et al., 2008) indicated that SW measures could be successfully applied but could lead to some disagreements when they are compared with more precise measurement solutions, suggesting that for reliable outcome two experienced raters should be involved in test administration. However, findings in our study revealed that SHT total time could be precisely measured by only one examiner, with sufficient familiarisation with the test procedure.

Our second finding is related to the minimum number of familiarisation trials necessary to obtain stable test outcome. It is well known that the number of familiarisation trials depends on a variety of factors where task complexity presents an important one (Moir et al., 2004; Moir et al., 2005; Glaister et al., 2007). In addition, possible strength gains may be a bias depending on the nature of task performed during the familiarisation process (Semmler, 2002; Kamen and Knight, 2004). Although contrast analysis revealed no significant differences between consecutive trials the differences in total time between the first and the last session clearly showed that the subjects tend to improve their results even after seven sessions. In addition, the trend within each session showed that the subjects are constantly improving their results, trial after trial. The differences between trials may be explained by neural adaptations, such as increasement in recruitment, firing rates, and synchronicity of motor units in agonist muscles, leading to strength gains (Semmler, 2002; Kamen and Knight, 2004). These adaptations occur particularly during the early phases of resistance training (Ritti-Dias et al., 2011; Nascimento et al., 2013). However, the differences between sessions could be related to the dependence of the adaptation rate on muscle size, with a larger muscle group requiring more sessions for familiarisation compared to a smaller group (Chan et al., 2020). Considering that SHT is a complex multi-joint task, large muscle groups are involved in performing the task. Due to the task complexity, prolonged familiarization is necessary to stabilize the results, i.e., the test should be performed with caution, when the aim is to assess the subject’s skill to perform consecutive medio-lateral jumps as fast as possible. However, there were no between leg differences in TT, indicating that in the healthy subjects, familiarization affects both legs similarly. This is in line with the finding of Itoh et al. (1998) who reported that more than 95% of the healthy subjects had normal between-leg differences. Other studies, reporting results for different variations of SHT in both legs of healthy patients also haven’t identified any notable between-leg differences (Docherty et al., 2005; Reid et al., 2007; Wikstrom et al., 2009; Kockum and Heijne, 2015). Further, LSI between dominant and nondominant leg calculated from total time was stable across both trials and sessions. This finding is particularly important, since the side hop test has been primarily introduced as a test aimed to detect potential asymmetries in lower leg function, particularly in patients recovering from ankle or knee injuries (Tegner et al., 1986; Risberg and Ekeland, 1994; Gustavsson et al., 2006; Caffrey et al., 2009; Wikstrom et al., 2009; Thomeé et al., 2011; Xergia et al., 2013). Consequently, these findings suggest that even within first session and few familiarizations’ trials, LSI could be assessed with consistency.

### Limitations

Present article has possible limitations that should be acknowledged: 1) although our sample size appear to be relatively small, but in fact, it was sufficient for the number of planned repetitions per subject (*k* = 7). For the alpha level of 0.05, power of 0.80, and 10% dropout rate, 12–14 subjects were required. 2) Only males were included into present study, therefore we cannot generalize results to both genders; 3) the resting time between sets could have also influenced the changes observed.

## Conclusion

Stopwatch measurements could be reliably used to measure total time in the SHT, while the test could be administrated with only one experienced rater. Although prolong familiarization is necessary for the subjects to stabilize the total time taken to perform given number of jumps as fast as possible, regardless of the leg, asymmetries assessed through the limb symmetry index are even within the first session. From the practical point of view, our findings confirmed that SHT could be easily administered by the practitioners without profound familiarization, while the potential asymmetries could be reliably assessed after only few familiarization trails.

## Data Availability

The original contributions presented in the study are included in the article/supplementary material, further inquiries can be directed to the corresponding author.
